# Effect of stellate ganglion block on postoperative recovery of gastrointestinal function in patients undergoing surgery with general anaesthesia: a meta-analysis

**DOI:** 10.1186/s12893-020-00943-0

**Published:** 2020-11-16

**Authors:** Bei Wen, Yajie Wang, Cong Zhang, Zhijian Fu

**Affiliations:** 1 Department of Pain Management, Shandong Provincial Hospital, Cheeloo College of Medicine, Shandong University, 324 Jingwu Road, Jinan, 250021 Shandong People’s Republic of China; 2 Department of Gastrointestinal Surgery, Shandong Provincial Hospital, Cheeloo College of Medicine, Shandong University, 324 Jingwu Road, Jinan, 250021 Shandong People’s Republic of China

**Keywords:** Stellate ganglion block, Gastrointestinal function, General anaesthesia, Postoperative recovery

## Abstract

**Background:**

The return of gastrointestinal function is an important sign of postoperative recovery in patients undergoing surgery with general anaesthesia. We aimed to summarize the effects of stellate ganglion block on the recovery of gastrointestinal function as a means of exploring methods through which anaesthesiologists can contribute to postoperative patient recovery.

**Methods:**

We performed a quantitative systematic review of randomized controlled trials published between January 1, 1988, and November 11, 2019, in PubMed, the Cochrane Library, China National Knowledge Infrastructure, Chinese VIP Information, and the Wanfang and SinoMed databases. Study quality was assessed by using the GRADE criteria and bias of included studies were assessed using the revised Cochrane risk-of-bias tool for randomized trials. The time to peristaltic sound resumption, flatus, postoperative eating and the incidence of abdominal bloating in the stellate ganglion block and control groups were compared. The control group consisted of either a stellate ganglion block with normal saline or no treatment. Meta-analysis was performed using Review Manager software.

**Results:**

After searching for relevant articles, 281 studies were identified, and five articles with data on 274 patients were eligible. Regarding postoperative flatus time, stellate ganglion block resulted in a mean reduction of 15 h (P = 0.02); then a sensitivity analysis was performed, and the standard mean difference decreased to 6 h (P = 0.007). For gastrointestinal surgery, the mean reduction was 23.92 h (P = 0.0002). As for the evaluation of the recovery of peristaltic sounds, stellate ganglion block promoted the recovery of regular peristaltic bowel sounds an average of 14.67 h earlier than in the control (P = 0.0008). When it comes to nutrients, stellate ganglion block shortened the total parenteral nutrition time by more than 50 h in patients who had undergone gastrointestinal surgery (P<0.00001). Finally, stellate ganglion block prevented the occurrence of postoperative abdominal bloating (P = 0.001).) No complications related to stellate ganglion block were reported.

**Conclusion:**

Stellate ganglion block may promote postoperative gastrointestinal recovery in patients undergoing various surgeries under general anaesthesia. However, additional trials investigating the use of stellate ganglion block are necessary to confirm our finding.

**Trial registration:**

This meta-analysis has been registered at the International Prospective Register of Systematic Reviews (registration number CRD42020157602).

## Background

The return of gastrointestinal (GI) function is an important sign of postoperative recovery in patients who have undergone surgery under general anaesthesia, especially in patients who receive abdominal surgery. With the emergence of *Enhanced Recovery After Surgery* (ERAS), the safe and effective promotion of functional GI function recovery plays an important role in rapid postoperative recovery and is an important consideration for both surgeons and anaesthesiologists.

Delayed recovery and postoperative disturbances of GI function prevent patients from resuming a normal diet and may lead to complications such as postoperative nausea and vomiting, abdominal distension and intestinal obstruction. Furthermore, it can also increase the incidence of anxiety and insomnia. These events could thus influence patients’ quality of life, prolong their hospital stays, increase the associated costs, and even increase the perioperative mortality rate [1]. A decrease in GI functional recovery is mainly attributable to 3 factors. (1) The first is functional changes in the autonomic nervous system, including excitation of the sympathetic system and inhibition of the parasympathetic system. Surgical trauma and stress enhance the activity of the hypothalamic–pituitary–adrenal (HPA) axis, resulting in the release of stress hormones such as catecholamine. These stress hormones can cause vasoconstriction in the digestive tract and destruction of the protective barrier [2, 3]. (2) The destruction and injury to normal GI structures, followed by inflammation, also delay postoperative GI recovery [4]. (3) The intraoperative and postoperative use of analgesics inhibits bowel function [5]. Furthermore, opioid usage can exacerbate GI dysfunction and delay GI recovery by acting peripherally [3].

Current methods to resolve this problem are conservative and include early ambulation, reduced opioid use, administration of intravenous fluids and antiemetics, and nasogastric tube placement; however, the effects of these interventions are sometimes limited. Since delayed postoperative recovery of GI function is often driven and exacerbated by heightened sympathetic tone, for anaesthesiologists, choosing appropriate anaesthesia, maintaining proper intraoperative management and applying appropriate interventions to prevent overexcitation of the sympathetic system are vital to prompt functional GI recovery in patients.

Stellate ganglion block (SGB) is currently the most commonly used sympathetic block in medical practice; it has a wide range of indications, including complex regional pain syndrome (CRPS) types 1 and 2, postherpetic neuralgia (PHN), intractable angina, post-traumatic stress disorder (PTSD), hyperhidrosis, arrhythmias, hot flushes, cerebrovascular disease and GI dysfunction [6, 7]. It can also modify the immune response and inhibit inflammation after acute trauma [8, 9]. Moreover, by blocking sympathetic nerves innervating the GI system, SGB can dilate GI vessels, improve the blood supply and enhance GI motility.

Based on this compelling rationale, some researchers have performed clinical trials to assess how SGB influences postoperative GI function. However, the sample sizes of these studies are relatively small, and the results are not completely consistent. It is difficult for an individual study to guide clinical practice. Thus, we sought to conduct a systematic review and meta-analysis of published studies exploring the effects of SGB on the recovery of GI function in patients undergoing surgery with general anaesthesia.

## Methods

This meta-analysis was conducted according to the Preferred Reporting Items for Systematic Reviews and Meta-Analyses (PRISMA) Statement [10] and has been registered at the International Prospective Register of Systematic Reviews (registration number CRD42020157602).

### Searching strategy

We searched for relevant clinical studies published between January 1, 1988 and November 11, 2019 by searching databases, including PubMed, Cochrane library, China National Knowledge Infrastructure (CNKI), Chinese VIP Information (VIP), the Wanfang and the SinoMed databases. Language was restricted to English and Chinese. All patients in these studies were adults > 18 years of age. Combined text and MeSH terms were used for searching; the detailed search strategies are described in Additional file 1: Appendix A. All potentially eligible studies were considered for review irrespective of the primary outcome. Manual searches were performed using the reference lists of crucial articles.

### Inclusion and exclusion criteria

Studies were considered eligible if they were random controlled clinical trials involving SGB for patients undergoing surgery with general anaesthesia and reporting postoperative GI function such as bowel sounds, the incidence of abdominal bloating, time to flatus, and time to eating. They should also include a control group, which was defined as patients receiving SGB with saline or general anesthesia alone (i.e. no block). The exclusion criteria were as follows: (1) observational and retrospective studies; (2) studies without a control group; (3) studies that did not assess GI function; (4) a reporting language other than English or Chinese.

### Study selection, data extraction and quality assessment

Study selection and data extraction were performed by three authors (BW, YJW, and CZ) independently. Disagreements and difficulties were resolved by group discussion or by consultation with another author (ZJF). The titles and abstracts of all articles were first screened according to the inclusion and exclusion criteria. The full text of the article was then carefully read for final determination. If the study satisfied the inclusion criteria, it was used for detailed analysis and data extraction. The following data were extracted from the selected studies: (1) demographic data (total number of participants, age, sex); (2) treatment protocols (methods, side treated, drug category, drug dosage); (3) the outcomes related with postoperative GI recovery (postoperative recovery time of bowel sounds, the incidence of abdominal bloating, time to flatus, and time to eating); and (4) any complications related with SGB. The bias of the included studies was assessed according to the revised Cochrane risk-of-bias tool for randomized trials (Rob-2). And the quality of trials was assessed using the GRADE system.

### Statistical analysis

We assessed the effect of SGB on postoperative GI recovery in terms of four outcomes: time to flatus, time to resumption of peristaltic sounds, postoperative eating, and incidence of abdominal bloating. The first of these was the primary outcome. The first three outcomes were analysed as continuous variables. The last outcome was analysed as a dichotomous variable. We reported absolute differences between patients who received different interventions and calculated pooled estimates of the mean differences in all these outcomes between the intervention groups. If the heterogeneity was low (I^2^ < 50%), a fixed-effects model was used for pooled analysis; a random-effects model was used if the heterogeneity was high to account adequately for the additional uncertainty associated with the use of data from different studies. Moreover, if the heterogeneity was high, a sensitivity analysis was performed to explore the cause of it. Publication bias was assessed using a funnel plot. We used the Cochran I^2^ test to assess the existence and magnitude of heterogeneity among the studies [11]. Heterogeneity was considered low, moderate, or high for I^2^ values < 25%, 25–50%, and > 50%, respectively.

Review Manager (RevMan 5.3) and Stata 14.0 was used in all statistical analyses. P ≤ 0.05 was considered statistically significant.

## Results

**Fig. 1:**
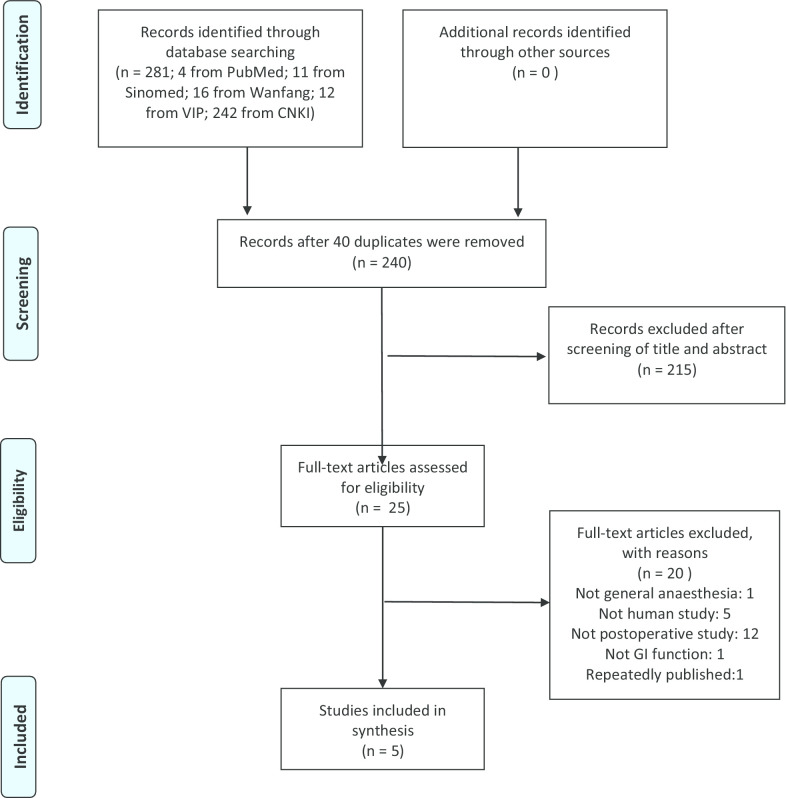
Flowchart of the study selection

In all, 281 studies were identified after a search for relevant articles; five articles [12–16] with data on 274 patients were eligible for this analysis. Of the 276 excluded studies, 41 were removed for duplications, 215 were removed after screening of their titles and abstracts according to the inclusion criteria, 1 was removed because the patient did not receive general anaesthesia, 5 were removed because they were not human studies, 12 were removed because they were not postoperative studies, 1 was removed because it did not focus on GI function, and 1 was removed because it was published repeatedly. Figure 1 shows the process of study selection.

### Summary characteristics of the included studies

The five studies included in our analysis that met the inclusion criteria but not the exclusion criteria were published between 2013 and 2019. The characteristics of the included studies are shown in Table 1, which lists the characteristics of these studies in detail, including type of operation, time and side of SGB, type and volume of local anaesthetic, and other parameters.

**Table 1:** Characteristics of the included studies

Study ID	Kind of operation	Age year (sd)	Sex M/F	Time of SGB	Ultrasound guided (yes or no)	Treatment of SGB group (n; side of placement; medication)	Treatment of control group (n; side of placement; medication)	Time of resuming peristaltic sound hours (sd)	Flatus time hours (sd)	Time of postoperative eating hours (sd)	Incidence of abdominal bloating	Harms related to SGB
Chunying et al. [13]	Laparoscopic gynecological surgery	43.57 (9.06)	0/41	After induction	Yes	21; right; 1% Lidocaine, 2 ml	20; right; 0.9% NS, 2 ml	SGB group: 22 (20.3) Control group: 30.3 (20.1)	SGB group: 15.4 (4.4); Control group: 20.7 (5.1)	–	SGB group: 23.8%; Control group: 60%	No
Lihua et al. [14]	GI surgery	57.69 (9.47)	41/14	Before induction	yes	18; left; 0.5% Ropivacaine, 7 ml	37; left; 0.9% NS, 7 ml	SGB group: 46 (31) Control group: 73 (36)	SGB group: 66 (34); Control group: 95 (45)	–	–	No
Renbo et al. [16]	GI surgery	62.07 (15.86)	43/35	Before induction	No	39; 1% Lidocaine, 8–10 ml	39; no treatment to SG	–	SGB group: 27.58 (6.7); Control group: 54.64 (8.9)	SGB group: 32.46 (6.3); Control group: 86.37 (9.5)	-	No
Peng et al. [17]	Posterior spinal surgery	43.60 (9.05)	21/19	After induction	Yes	20; right; 1% Lidocaine, 6 ml	20; right; 0.9% NS,6 ml	SGB group: 16.2 (24.9); Control group: 32.1 (24.8)	SGB group: 12 (4.4); Control group: 14.7 (4.6)	–	SGB group: 10%; Control group: 45%	No
Yuxin et al. [15]	GI surgery	57.45 (8.12)	48/12	Before induction	no	30; right; 1% Lidocain, 8–10 ml	30; no treatment to SG	–	SGB group: 72.15 (17.1); Control group: 85.8 (20.5)	SGB group: 179.8 (41.7); Control group: 229.35 (83.6)	-	No

Data are presented as mean ± SD. Items that could not be extracted from the original articles are described as “−”

*SGB* stellate ganglion block, *GI* gastrointestinal, *NS* normal saline, *h* hours

### Risk of bias of included studies

**Fig. 2:**
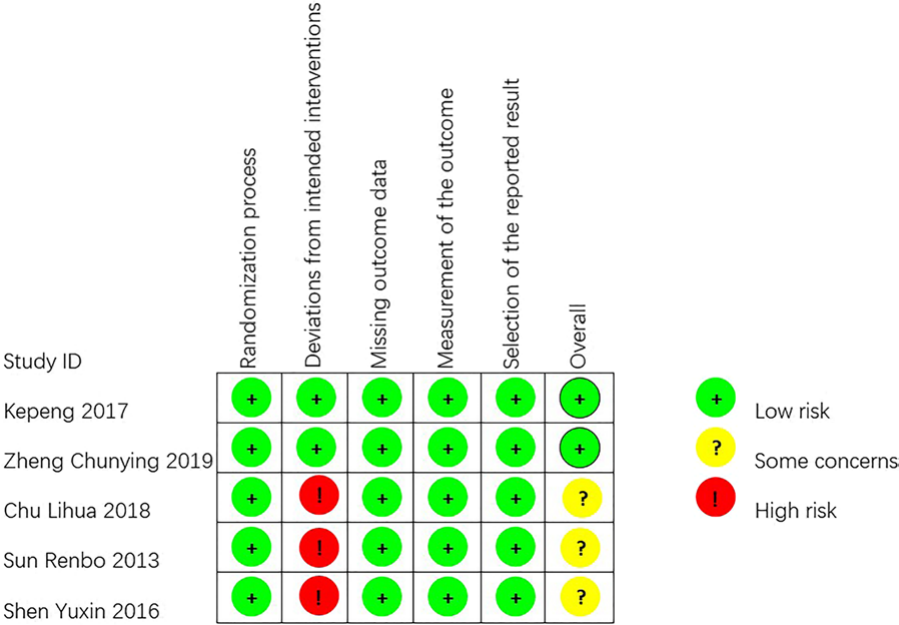
Bias assessment graph. Bias were assessed by using the Rob2 tool

**Fig. 3:**
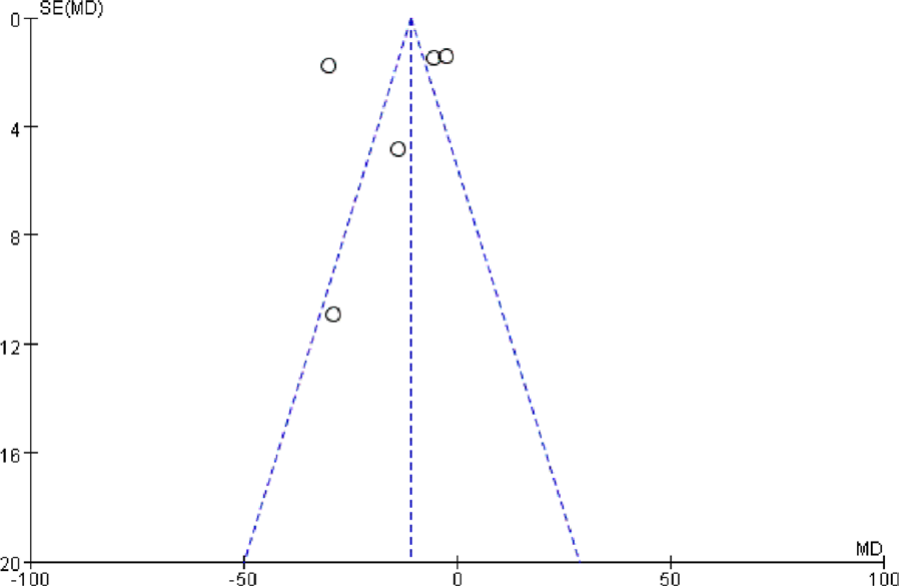
Funnel plot for postoperative flatus time. SE, standard error; MD, mean difference. The blue vertical dashed line represents the mean of MD, and the 2 blue slant dashed lines represent 95% CI of MD

The bias of the included studies was assessed according to the Rob-2 [17]. Figure 2 presents detailed information about this assessment. Two studies had low overall bias [12, 16], and three had some concerns regarding bias [13–15]. All three of these studies performed SGB before induction and relied on the observation of Horner’s syndrome to ensure a successful block; thus, the care givers and those delivering the interventions might have been aware of the participants’ assigned interventions. In two of the included studies, no sham procedures were performed, so the allocation concealment and blinding of participants and personnel could not be assessed [14, 15]. Figure 3 shows a funnel plot of the included studies. Because we included only 5 studies, quantitative analyses such as the Begg and Egger tests were not performed. The overall quality of our meta-analysis is shown in Table 2; the results indicate that we have a moderate overall certainty of evidence.

**Table 2:** Quality of assessment of primary outcome according to the GRADE system

Certainty assessment							Summary of findings				
Participants (studies) Follow up	Risk of bias	Inconsistency	Indirectness	Imprecision	Publication bias	Overall certainty of evidence	Study event rates (%)		Relative effect (95% CI)	Anticipated absolute effects	
							With sham therapy or blank control	With SGB		Risk with sham therapy or blank control	Risk difference with SGB
Postoperative flatus time (Scale from: 0 to 100)											
274 (5 RCTs)	Not serious ^a^	Serious^b^	Not serious	Not serious ^c^	None^d^	⨁⨁⨁**◯** Moderate	146	128	–	The mean postoperative flatus time was **146** h	MD **15.07 h lower** (27.58 lower to 2.56 lower)

*CI* confidence interval, *MD* mean difference

^a^Four studies reported how the random sequence was generated, while one only stated that patients were randomly allocated into two groups but did not mention how randomization. Two included studies did not perform any sham procedures, so the allocation concealment and blinding of participants and personnel could not be assessed, leading to a high risk of selection and performance bias [15, 16]. Furthermore, no studies described blinding of outcome assessments

^b^The number of included studies is small, and there are some differences among them

^c^We calculate the optimal information size, the largest one is 102, and the total sample of each group are both more than 102

^d^The publication bias was assessed by using funnel plot (the funnel plot is symmetrical), the Begg and the Egger test (the P value were 0.22 and 0.65 separately)

### Primary outcome

**Fig. 4:**
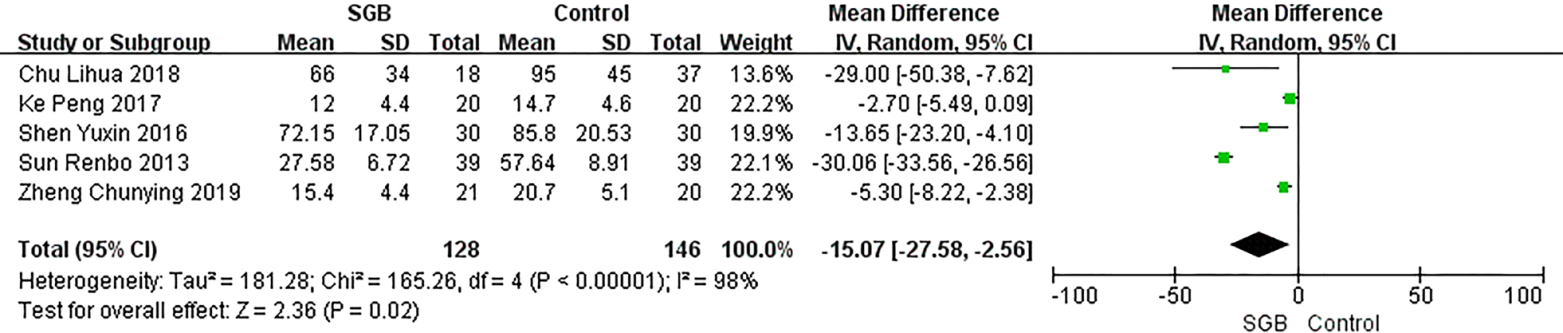
Postoperative flatus time. *CI* confidence interval, *SD* standard error, *SGB* stellate ganglion block. Postoperative flatus time in patients receiving SGB with local anesthetics compared to control in different surgeries

**Fig. 5:**
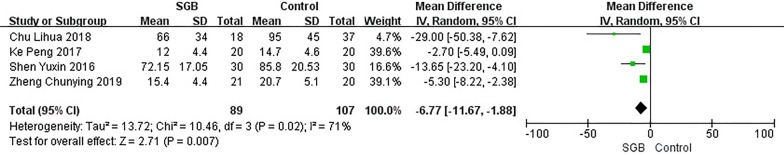
Postoperative flatus time after sensitive analysis. *CI* confidence interval, *SD* standard error, *SGB* stellate ganglion block. Postoperative flatus time in patients receiving SGB with local anesthetics compared to control after excluding a heterogeneity-causing study

Our primary outcome is comparison of the postoperative flatus time in the SGB and control groups; this comparison is shown in Fig. 4. All included studies reported this outcome, but there was great heterogeneity (P < 0.00001, I^2^ = 98%). Our analysis showed an overall effect size (mean difference, MD) of − 15.07 h (95% CI − 27.58, 2.56) with a Z value of 2.36 (P = 0.02 < 0.05). However, as shown in Fig. 5, when we deleted one particular study [15], the heterogeneity was greatly reduced (P = 0.02 < 0.05, I^2^ = 71%), and the overall effect size (MD) became − 6.77 h (95% CI: − 11.67, 1.88) with a Z value of 2.71 (P = 0.007 < 0.05). We think that this phenomenon was caused by the low quality of that study, as shown in Figs. 1 and 2; that study did not describe how the random sequence was generated, the SG did not receive any treatment in the control group, and the side on which SGB was performed in the SGB group was not reported. Furthermore, the population evaluated in this study was the oldest among the five studies, and the surgery was performed to treat GI tumours. However, despite the heterogeneity, inclusion of that study did not change the effect of SGB on the postoperative flatus time.

**Fig. 6:**
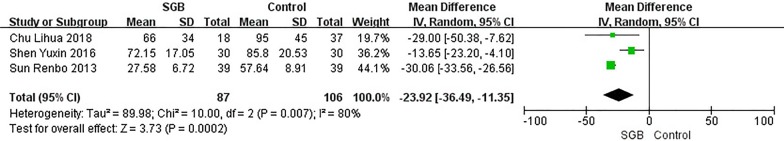
Postoperative flatus time in GI surgery. *CI* confidence interval, *SD* standard error, *SGB* stellate ganglion block. Postoperative flatus time in patients receiving SGB with local anesthetics compared to control in GI surgery

We also performed a subgroup analysis of 3 clinical trials in patients who underwent GI surgery surgery [13–15]. In these three studies, SGB was performed before induction of general anaesthesia. As shown in Fig. 6, the overall effect size (MD) was − 23.92 h (95% CI − 36.49, 11.35) with a Z value of 3.73 (P = 0.0002 < 0.05), indicating that administration of SGB before anaesthesia significantly shortened the time to flatus after GI surgery.

Overall, our analysis suggests that SGB may shorten the postoperative flatus time in patients who undergo various types of surgery under general anaesthesia, with a mean reduction of more than 6 h. This reduction was most obvious in patients who underwent GI surgery, with a mean reduction of 23.92 h.

### Secondary outcomes

#### Time to resumption of peristaltic sounds

**Fig. 7:**
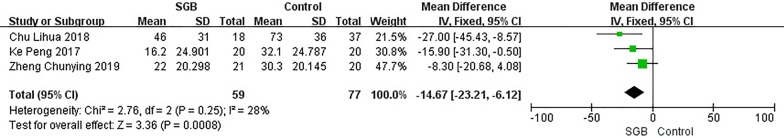
Time to resuming postoperative peristaltic sound. *CI* confidence interval, *SD* standard error, *SGB* stellate ganglion block. Time to resuming postoperative peristaltic sound in patients receiving SGB with local anesthetics compared to control

Three of the included studies reported the time to resumption of peristaltic sounds after surgery; these studies involved patients who underwent GI, laparoscopic gynaecological and posterior spinal surgery, respectively [12, 13, 16]. However, the data were presented as the number of patients whose bowel sounds recovered within a given period of time after the operation, such as before 12 h, before 24 h, before 36 h, before 48 h, and before 72 h postoperatively. For convenience, we converted these data as follows: the average of the reported times was used as the time to resumption of peristaltic sounds in the patients; thus, this outcome was also analysed as continuous. Figure 7 shows a comparison of postoperative peristaltic sound resumption time in the SGB and control groups. The overall effect size (MD) was − 14.67 h (95% CI − 23.21, − 6.12) with a Z value of 3.36 (P = 0.0008 < 0.05). Furthermore, the heterogeneity was low (P = 0.25 > 0.05, I^2^ = 28%), indicating that the combined analysis of these three studies is reasonable. This outcome, to some degree, may show that SGB can promote GI movement after different types of surgery in patients who receive surgeries under general anaesthesia. However, additional studies are needed to confirm this.

#### Time to postoperative eating

**Fig. 8:**
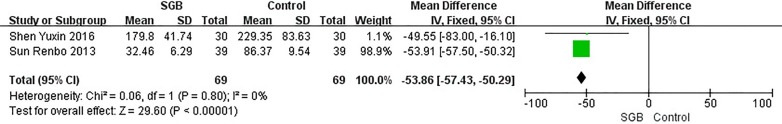
Time to postoperative eating. *CI* confidence interval, *SD* standard error, *SGB* stellate ganglion block. Time to postoperative eating in patients receiving SGB with local anesthetics compared to control

Two of the included studies reported time to postoperative eating, and the parameters in these two studies were very similar [14, 15]. First, both studies involved patients who had undergone GI surgery. Second, SGB was performed before the induction of general anaesthesia in both studies. Third, the local anaesthetic used consisted of 8–10 ml of 1% lidocaine in both studies. Last, neither study used a sham group, and no treatment was applied in the control group. Figure 8 shows the data for this outcome. There was no heterogeneity between these 2 studies (P = 0.80 > 0.05, I^2^ = 0%). The overall effect size (MD) was − 53.86 h (95% CI − 57.43, − 50.29) with a Z value of 29.60 (P < 0.00001). This result suggests that in GI surgery under general anaesthesia, performing SGB before the induction of anaesthesia can significantly shorten the time to postoperative eating by more than 2 days.

#### Incidence of postoperative abdominal bloating

**Fig. 9:**
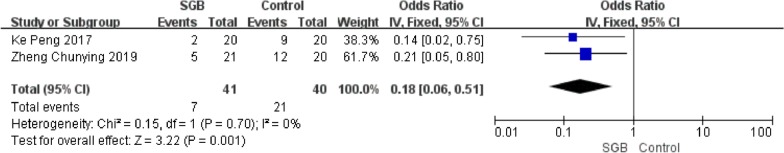
Incidence of postoperative abdominal bloating. *CI* confidence interval, *SD* standard error, *SGB* stellate ganglion block. Incidence of postoperative abdominal bloating in patients receiving SGB with local anesthetics compared to control

Two studies included data on the incidence of postoperative abdominal bloating [12, 16]. One study involved laparoscopic gynaecological surgery [12], and the other involved GI surgery [16]. As shown in Fig. 9, no heterogeneity was found (P = 0.7070 > 0.05, I^2^ = 0%), and odds ratio, OR) was 0.18 (95% CI 0.06, 0.51) with a Z value of 3.22 (P = 0.001 < 0.05). The result indicates that SGB may reduce the incidence of postoperative abdominal bloating.

## Discussion

With the continuously increasing incidence of diseases with surgical indications, the number of patients who require general anaesthesia is also increasing. Both surgical manipulation and analgesia can lead to postoperative GI dysfunction. Normally, after GI surgery, gastric motility recovers in 24–48 h, small intestinal motility recovers in 12–24 h, and colonic motility recovers in 3–5 days [18]. The inhibition of GI function can bring about GI dysfunction and discomfort; more seriously, it could lead to systematic inflammation and even to multiple organ dysfunction syndrome [19].

There have been many studies aiming to explore methods to facilitate postoperative GI recovery. These studies have explored the following methods: (1) multimodal analgesia to reduce the use of opioids, e.g., other analgesic methods and the use of nonsteroidal anti-inflammatory drugs (NSAIDs) [20, 21]; (2) laparoscopic surgery [22]; (3) goal-directed fluid therapy [22]; (4) early enteral nutrition [23]; (5) gum chewing [24]; (6) the use of opioid receptor antagonists [25]; and (7) traditional Chinese medicine [26]. All of these methods have been shown to have limited effects in postoperative GI function recovery.

To determine whether SGB plays a role in postoperative GI function, we performed the meta-analysis reported in this paper. Although the included studies involved patients who underwent various types of surgery, the results nevertheless suggest that SGB may promote postoperative GI recovery in patients who underwent surgery with general anaesthesia. Our results show that SGB caused a mean reduction of 15 h in the time to flatus after different surgeries; after a study that caused heterogeneity was excluded, the mean reduction was still greater than 6 h. Further analysis of only patients who underwent GI surgery showed a mean reduction of 23.92 h. The data on the recovery of peristaltic sounds suggested that in patients who receive various types of surgeries, SGB promotes the recovery of regular bowel sounds 14.67 h earlier, on average, than the time observed for the control group. Regarding nutrients, the use of SGB in GI surgery can shorten the time for which total parenteral nutrition is required by more than 50 h. Furthermore, our study shows that SGB can prevent the occurrence of postoperative abdominal bloating. None of the included studies reported complications related to SGB.

The role played by SGB on postoperative recovery might be explained by the following mechanism. The digestive system is mainly governed by the autonomic nervous system. As a major stressor, undergoing surgery with general anaesthesia leads to functional changes in this system, causing stimulation of the sympathetic system, inhibition of the parasympathetic system and the release of catecholamines. Blockage of cervical sympathetic nerves can suppress the overexciting of the sympathetic nervous system, help to establish balance of the autonomic nervous system and promote the establishment of homeostasis via regulation of the neuroendocrine-immune system [27], thus promote postoperative recovery of GI function.

As the most commonly used cervical ganglion block, SGB can be applied with or without imaging guidance through computed tomography or ultrasound [28, 29]. Manifestations including Horner’s syndrome, an increase in skin temperature, loss of the galvanic skin response and an increase in blood flow in the innervated areas, indicate success of the block. With the popularization of ultrasound, performing SGB under ultrasound guidance, which provides direct visualization of soft tissue structures around the sympathetic chain, appears to offer increased safety and efficacy [28]. This allows more effective and precise needle placement using a small volume of drugs. Administration of a series of local anaesthetics such as lidocaine, bupivacaine and ropivacaine can be chosen for reversible blockade. Moreover, neurolytic agents such as alcohol can be used for permanent blockade. If SGB is performed under the guidance of ultrasound, 2–5 ml of drug is required to ensure that the procedure is as selective for sympathetic block as possible [28]. When ultrasound guidance is not possible, it has been reported that 6 to 10 ml of drug is needed to achieve a successful blockade [30–32]. SGB was performed after and before the induction of anaesthesia in two and three studies, respectively. Performing SGB before induction can provide visible evidence of the success of the block, for example if the patient presents Horner’s syndrome, but prevents blinding of the patients and doctors, while performing SGB after induction allows blinding but increases the difficulty of assessing the block’s efficacy. However, if the operator is skilled or can perform SGB under ultrasound guidance, performing SGB after induction is theoretically a good choice.

There are some limitations to our study. First, only 5 studies were included in our analysis, limiting the subgroup analysis. These five studies involved patients who underwent 3 different types of surgery, and each type of surgery was addressed in only 1 or 3 studies; thus, additional studies should be performed to validate our conclusions. Second, all of the clinical trials included in this study were conducted in the People’s Republic of China, restricting the generalizability of our conclusions. Moreover, SGB is an invasive treatment, and success of the block needs to be verified by some manifestations, making blinding impossible.

## Conclusion

The quantitative analysis presented here shows that SGB may be moderately effective in promoting GI recovery in patients who have undergone surgery. It could reduce postoperative flatus time, promote the recovery of peristaltic sounds, shorten the total parenteral nutrition time after GI surgery and prevent the occurrence of postoperative abdominal bloating. Further clinical trials of high quality and the use of SGB in other types of surgery and in other countries are needed to confirm these results.

## Supplementary information

**Additional file 1:** Searching strategy in PubMed database.

## Data Availability

All data generated or analysed during this study are included in this published article.

## Abbreviations

SGB: Stellate ganglion block
GI: Gastrointestinal
ERAS: Enhanced recovery after surgery
SG: Stellate ganglion
CRPS: Complex regional pain syndrome
PHN: Postherpetic neuralgia
PTSD: Post-traumatic stress disorder
PRISMA: Systematic reviews and meta-analyses
CNKI: China National Knowledge Infrastructure
VIP: Chinese VIP Information
