# Supplementary material for: Effect of stellate ganglion block on postoperative recovery of gastrointestinal function in patients undergoing surgery with general anaesthesia: a meta-analysis
Source: BMC Surg. 2020 Nov 16;20:284. doi: 10.1186/s12893-020-00943-0 (PMC7670678; doi:10.1186/s12893-020-00943-0)
Supplement: Supplementary file 1 — Additional file 1: Searching strategy in PubMed database. [file 12893_2020_943_MOESM1_ESM.docx]

**PubMed**

“satellite ganglion” and “gastrointestinal function”. The complete search strategy used for PubMed was:(( ( Stellate Ganglion [Mesh] OR Ganglion, Stellate [Title/Abstract] OR Cervicothoracic Ganglion [Title/Abstract] OR Ganglion, Cervicothoracic [Title/Abstract] OR Cervicothoracic Ganglia [Title/Abstract] OR Ganglia, Cervicothoracic [Title/Abstract] OR Stellate Ganglia [Title/Abstract] OR Ganglia, Stellate [Title/Abstract] OR Ganglias, Stellate [Title/Abstract] OR Stellate Ganglias [Title/Abstract]) OR (Ganglia, Sympathetic [Mesh] OR Ganglion,Sympathetic [Title/Abstract] OR Sympathetic Ganglion [Title/Abstract] OR Sympathetic Ganglia [Title/Abstract] OR Celiac Ganglia [Title/Abstract] OR Ganglia, Celiac [Title/Abstract] OR Celiac Ganglion [Title/Abstract] OR Ganglion, Celiac [Title/Abstract]) ) AND (Gastrointestinal function [Title/Abstract] OR bowel sounds [Title/Abstract] OR flatulence [Title/Abstract] OR flatus time [Title/Abstract] OR exhaust time [Title/Abstract])))
